# The risk for inflammatory joint disease in patients with severe or treatment-resistant depression: population-based cohort study in Sweden

**DOI:** 10.1192/j.eurpsy.2024.1110

**Published:** 2024-08-27

**Authors:** P. Brenner, J. Askling, D. Hägg, L. Brandt, P. Stang, J. Reutfors

**Affiliations:** ^1^Karolinska Institutet, Stockholm, Sweden; ^2^Janssen Research and Development, (retired), Titusville NJ, United States

## Abstract

**Introduction:**

Inflammatory joint diseases (IJD), including rheumatoid arthritis (RA), psoriatic arthritis (PsA), ankylosing spondylitis/spondyloarthropathies (AS), and juvenile idiopathic arthritis (JIA), are more common in patients with depression. However, it remains unclear whether the strength of this association varies with the severity or level of treatment resistance of the depressive episode.

**Objectives:**

To assess the risk for IJD in patients with severe depression and TRD compared to population comparators and patients with non-severe and non-treatment resistant depression.

**Methods:**

We conducted parallel cohort studies among 600,404 patients with a depressive episode identified in Swedish nationwide administrative registers. The prospective risk for IJD, both overall and per IJD condition, in patients with depression of any severity was compared to matched population comparators. Additionally, we assessed the same associations comparing patients with depression to those with severe or treatment-resistant depression. Analyses were adjusted for comorbidities and sociodemographic covariates.

**Results:**

Overall, patients with depression were at increased risk for later IJD compared to population comparators (adjusted hazard ratio (aHR) for any IJD 1.34 [95% CI 1.30-1.39]; RA 1.27 [1.15-1.41]; PsA 1.45 [1.29-1.63]; AS 1.32 [1.15-1.52]). The associations were not significantly different for patients with severe depression or TRD.

**Conclusions:**

Patients with severe and treatment resistant depression are at higher risk for inflammatory joint disease than population comparators. This association does not seem to be stronger than for patients with non-severe or non-resistant depression. Severity and treatment resistance of a depressive episode as identified in register data may not be valid depressive phenotypes for predicting risk for inflammatory joint disease.

**Disclosure of Interest:**

P. Brenner Grant / Research support from: Affiliated with/employed at the center for Pharmacoepidemiology, Karolinska Institutet, which receives grants from several entities (pharmaceutical companies, regulatory authorities, contract research organizations) for the performance of drug safety and drug utilization studies., J. Askling Grant / Research support from: Karolinska Institutet has entered into agreements with the following companies, with JA as PI: Abbvie, BMS, Eli Lilly, Galapagos, Janssen, Pfizer, Roche, Samsung Bioepis and Sanofi., D. Hägg Grant / Research support from: Affiliated employed at the center for Pharmacoepidemiology, Karolinska Institutet, which receives grants from several entities (pharmaceutical companies, regulatory authorities, contract research organizations) for the performance of drug safety and drug utilization studies., L. Brandt Grant / Research support from: Affiliated with/employed at the center for Pharmacoepidemiology, Karolinska Institutet, which receives grants from several entities (pharmaceutical companies, regulatory authorities, contract research organizations) for the performance of drug safety and drug utilization studies., P. Stang Employee of: Former employee of Janssen Research & Development, LLC. The work on this study was part of the employment., J. Reutfors Grant / Research support from: employed at the center for Pharmacoepidemiology, Karolinska Institutet, which receives grants from several entities (pharmaceutical companies, regulatory authorities, contract research organizations) for the performance of drug safety and drug utilization studies.

